# Comparative evaluation of decellularized bovine omentum alone and in combination with mitomycin-C in the management of corneal injuries in dogs

**DOI:** 10.14202/vetworld.2020.2401-2410

**Published:** 2020-11-11

**Authors:** A. S. Thajunnisa, Anoop Sainulabdeen, K. M. Dileepkumar, Laiju M. Philip, V. N. Vasudevan, C. B. Devanand

**Affiliations:** 1 Department of Veterinary Surgery and Radiology, College of Veterinary and Animal Sciences, Kerala Veterinary and Animal Sciences University, Mannuthy, Kerala, India; 2 Department of Livestock Products Technology, College of Veterinary and Animal Sciences, Kerala Veterinary and Animal Sciences University, Mannuthy, Kerala, India

**Keywords:** biomaterial scaffold graft, corneal ulcer, decellularized bovine omentum, graft assisted corneal healing, mitomycin-C

## Abstract

**Background and Aim:**

Ulcerative corneal lesions are common ocular affections encountered in veterinary ophthalmology, having a higher incidence in dogs with brachycephalic conformation. Prompt and effective diagnosis and repair are necessary to avoid corneal perforation and restore vision. Corneal wound healing is a complex phenomenon often resulting in vision impairment as a consequence of corneal fibrosis and pigmentation. The present study investigated the efficacy of decellularized and gamma-irradiated bovine omentum as an extracellular matrix scaffold in the reconstruction of extensive and full-thickness corneal defects, and the cytotoxic effects of mitomycin-C (MMC) to prevent corneal fibrosis and pigmentation.

**Materials and Methods:**

Twelve injured corneas of eleven dogs irrespective of breed, age, and sex were randomly divided into Groups I and II, consisting of six corneas each. Under general anesthesia, corneal grafting with decellularized and gamma-irradiated bovine omentum was carried out in Group I, whereas Group II corneas underwent single time intra-operative application of topical MMC for 2 min before corneal grafting with the same material. Epithelialization of cornea and observations including corneal edema, neovascularization, the extent of pigmentation, corneal clarity, and scarring was recorded on days 7, 14, 21, and 60 postoperatively.

**Results:**

All corneas in Group I showed early epithelialization by day 7 compared to Group II where the MMC delayed epithelialization in 50% of the corneas. Visual function scores improved greatly from 0.17±0.17 in Group II on the day of presentation to 1.0±00 by the end of the observation period compared to Group I (from 0.33±0.15 to 0.88±0.11). Although epithelialization and corneal healing were delayed, 50% of the corneas recovered with undetectable corneal scar and melanosis at the end of the observation period in Group II due to the anti-fibrotic effect of MMC.

**Conclusion:**

From the present study, it was concluded that re-epithelialization of the cornea was enhanced by corneal grafting with decellularized bovine omentum, and application of MMC was effective in delaying corneal fibrosis and pigmentation.

## Introduction

Ulcerative corneal lesions are common and important ocular conditions in dogs. Prompt and effective repair of large diameter and full-thickness corneal defects is recommended to avoid corneal perforation and restore vision. Although medical therapy is adequate for the management of superficial epithelial defects, descemetoceles and full-thickness lacerations are indications for emergency surgical interventions [1]. Several grafting techniques have been described with varying efficacy for the replacement of lost corneal substance. This includes lamellar corneal grafting, corneoscleral transposition, penetrating keratoplasty, conjunctival grafts including island, pedicle, bulbar, bridge, advancement, or complete bulbar graft, as well as synthetic grafts and biomaterial grafts [2,3]. Loss of corneal clarity due to scarring and melanosis are the frequent complications encountered with all of these techniques. Penetrating keratoplasty or corneal transplantation has proven effective with no to minimal complications and absolute clarity [4]. However, the lack of an animal eye bank and the difficulty in obtaining fresh or frozen corneal transplants limits its application in veterinary ophthalmology. Hence, trials were done with amniotic membrane, porcine small intestinal submucosa, porcine urinary bladder submucosa, and porcine cholecyst [5-7].

Decellularized and gamma-irradiated bovine omentum is a novel biological scaffold for corneal surface reconstruction. Preservation of three-dimensional architecture of connective, elastic, and reticular fibers and glycosaminoglycans potentiates recellularization and is expected to enhance corneal healing [8]. Corneal fibrosis is a common consequence of various canine keratopathies, and ulcer repair often results in significant visual impairment. To date, therapies specifically targeting canine corneal fibrosis are limited in veterinary ophthalmology. The cytotoxic and anti-mitotic effects of mitomycin-C (MMC) were observed to be beneficial in controlling myofibroblast proliferation and, thus, corneal scarring [9].

The present study investigates the efficacy of decellularized and gamma-irradiated bovine omentum as an extracellular matrix scaffold in the reconstruction of extensive and full-thickness corneal defects, and the cytotoxic effects of MMC to prevent corneal scarring. Hence, the study was carried out to evaluate the efficacy of decellularized and gamma-irradiated bovine omentum alone and in combination with MMC as a collagen graft for the treatment of corneal injuries in dogs.

## Materials and Methods

### Ethical approval

This study was approved by the Institutional Animal Ethics Committee, College of Veterinary and Animal Sciences, Mannuthy, Kerala, India, vide order no KVASU/DAR/Acad A1/14531/2017.

### Animals and experimental design

The study was conducted on 12 corneas from 11 dogs with deep corneal injuries including corneal perforations/staphyloma, melting ulcers, and descemetocele that presented to the Teaching Veterinary Clinical Complex, College of Veterinary and Animal Sciences, Mannuthy, Kerala, India during March to December 2018. On the day of presentation, each animal was randomly assigned to one of the two groups (n=6) after a detailed clinical and ophthalmic examination. The corneas of animals in Group I (C1 to C6) underwent reconstruction with decellularized bovine omental scaffold graft and animals in Group II (C7 to C12) received a single time intra-operative application of topical MMC (0.02% for 2 min) before grafting.

### Surgical management

Signalment and detailed history of the duration of illness and previous treatment regimen, if any, were recorded for each case. All animals underwent detailed clinical evaluation for general body condition, physiological parameters (rectal temperature, the color of visible mucous membrane, respiratory rate, and pulse rate), routine hematology, random blood sugar, and detailed ophthalmic examinations including ocular signs, the appearance of adnexa, visual function tests, presence of corneal edema, corneal clarity, neovascularization, measurement of tear production by Schirmer tear test 1, evaluation of depth and layer of cornea affected by fluorescein dye test (FDT), tonometry, direct and indirect ophthalmoscopy, slit-lamp biomicroscopy, antibiogram of corneal swabs, and impression cytology.

Decellularization of the raw bovine omentum was performed using a natural biological detergent following a validated proprietary protocol and sterilized by gamma irradiation (25 KGy). Ten-x 10 cm^2^ defatted, decellularized, and gamma-irradiated bovine omentum were procured, after all profile testing, from the Department of Livestock Products Technology, College of Veterinary and Animal Sciences, Mannuthy, Kerala, India. Injectable MMC in powder form available as 2 mg vials was reconstituted with normal saline to prepare a 0.02% solution for topical application in Group II animals.

All animals underwent pre-anesthetic fasting and the procedure was carried out under general anesthesia. Dogs were pre-medicated with atropine sulfate (0.045 mg/kg body weight dosing) and xylazine hydrochloride (1 mg/kg) administered intramuscularly, and general anesthesia was induced with ketamine hydrochloride (5 mg/kg) and midazolam (0.1 mg/kg) administered intramuscularly. Anesthesia was maintained with 2% isoflurane after endotracheal intubation. Aseptic preparation of the peri-ocular region was achieved with 5% Povidone-iodine solution. The diameter of lesions was measured in millimeters using the STT strip and the area was calculated (*A=*π*r^2^*) to record the lesion. The decellularized bovine omental transplant was prepared so that its size was consistently 1-2 mm larger than the area of corneal defect. The size matched graft was hydrated in sterile normal saline for 5 min before grafting. The superficial cornea was debrided to remove loose epithelium and other tissue debris using corneal scissors and sterile cotton tip applicators. The size matched rehydrated transplant was placed over the graft bed and retained in position with simple interrupted sutures at four cardinal points around the corneal defect using monofilament polycryl, (polyglycolic acid 910, size 10/0, 8/0, or 6/0). The suturing was then completed with an appropriate number of sutures in each case. Liberal irrigation with normal saline was performed throughout the procedure to avoid drying of the cornea. In Group II corneas, a single time intra-operative application of 0.02% MMC was carried out with a sterile cotton swab, left in place for 2 min, and thoroughly washed off with normal saline before fixing the graft. Temporary tarsorrhaphy was performed in all the cases and retained until day 7, and Elizabethan collar was advised to avoid self-mutilation. Systemic antibiotic therapy, topical instillation of antibiotic (moxifloxacin), anti-inflammatory, cycloplegic, and hyperosmotic eye drops were followed on case to case bases depending on anticipated complications.

### Post-operative evaluation

Post-operative observations were recorded on days 7, 14, 21, and 60. Ophthalmic examination included visual function evaluation (evaluated based on scores allotted to menace response, cotton ball testing, and pupillary light response), STT, FDT, direct and indirect ophthalmoscopy, and slit-lamp biomicroscopy. The cornea was graded for corneal clarity, edema, neovascularization, extent of corneal pigmentation/melanosis, and intensity of corneal scar. Corneal clarity was recorded as clear (4+), hazy (3+), moderate opacity (2+), or complete opacity (1+) [10]. Corneal edema was graded as no edema (0), mild (1), marked (2), or severe edema (3) [11]. Corneal neovascularization was graded as no visible vessels (0), mild superficial vascularization (1), profuse superficial vascularization (2), or extensive vascularization with vessels originating from all the quadrants of the cornea (3). The cornea was schematically divided into 24 sectors for documentation and each corneal sector was evaluated for the extent of pigmentation [11,12]. For grading of corneal scars, the cornea was allocated into one of five categories (Table-1) based on the opacity of corneal scar, presence of ghost vessels, and the ability to visualize the posterior and/or anterior segment through the grafted site on final follow-up [7]. Intraocular pressure was measured using applanation tonometry on days 21 and 60 post-grafting, once epithelialization was complete, to avoid possible irritation to the healing cornea.

**Table-1:** Grading of corneal fibrosis/scarring at the end of observation period (day 60) in Group I and Group II animals.

Grade	Category	Description	Cornea	No. of corneas		Per cent	
	
				Group I	Group II	Group I	Group II
0	No opacity	Almost undetectable corneal opacity, ghost stromal vasculature, and clear visualization of the posterior segment through the graft	C1, C8, C9, C12	1	3	8.33	33.33
1	Minimum opacity	Minimal stromal opacity and vascularization, clear visualization of the posterior segment through the graft	C2, C3, C11	2	1	16.6	8.33
2	Mild to moderate opacity	Mild-to-moderate stromal opacity and vascularization. Visualization of the posterior segment through the graft is possible, but difficult	C7, C10	0	2	0	16.6
3	Marked opacity	Marked stromal opacity and vascularization allowing visualization of the anterior chamber through the graft, but not the posterior segment	C4, C5	2	0	16.6	0
4	Severe opacity	Severe stromal opacity, vascularization, and pigmentation. Anterior chamber cannot be visualized through the graft	C6	1	0	8.33	0

C=Cornea

### Statistical analysis

Data were analyzed using SPSS version 24.0 (IBM Corp., USA). The tests adopted were paired t-test, repeated analysis of variance, Friedman’s test, Mann–Whitney test, and Wilcoxon test, depending on the variables and comparison required. The level of significance was fixed at 5% (p<0.05).

## Results

### General observations

The general observations of the animals used for the study are presented in Table-2. Out of eleven dogs selected, five were Chinese pugs (45.45%), three were French Bulldogs (27.27%), two were Pitbulls (18.18%), and one was a Labrador Retriever (9.09%). The age of dogs included in the study ranged from 3 months to 38 months, with mean values of 19.83±4.82 months in Group I and 7.67±2.0 months in Group II. Among 11 dogs, six were male and five were female; right and left corneas were affected in equal proportion. All animals included in the study had good general body condition, and physiological and hematological parameters did not show any significant differences within or between the groups during the observation period.

**Table-2:** List of animals in the study with general observations on breed, age, sex, eye affected, type of lesion, area of lesion, duration since occurrence and complications of condition in Group I and Group II.

Group	Cornea No.	Breed	Age (months)	Sex	Eye affected	Type of lesion	Size of the defect (mm)	Area of lesion (mm^2^) (*A=πr^2^*)	Duration since occurrence	Complications
I	C1	Pitbull	38	Female	Left	Descemetocele	13	132.66	6 days	Nil
	C2	Chinese Pug	18	Male	Right	Staphyloma	6	28.26	3 days	Iris Prolapse
	C3	Chinese Pug	24	Male	Left	Staphyloma	4	12.56	7 days	Iris prolapse Miosis
	C4	Pitbull	4	Male	Right	Keratomalacia	8	50.24	8 days	Melting keratitis
	C5	Chinese Pug	24	Female	Left	Staphyloma	6	28.26	4 days	Iris prolapse Melting margins, Miosis
	C6	Chinese Pug	11	Male	Right	Keratomalacia	10	78.5	8 days	Melting keratitis
II	C7	French Bulldog	8	Female	Right	Staphyloma	10	78.5	5 days	Nil
	C8	Labrador	5	Female	Left	Dermoid		32	Congenital	Nil
	C9	French Bulldog	10	Female	Left	Staphyloma	9	63.58	5 days	Nil
	C10	Pug	3	Male	Right	Staphyloma	12	113.04	4 days	Iris prolapse
	C11	French Bulldog	16	Male	Right	Staphyloma	9	63.58	3 days	Nil
	C12	Pitbull	4	Male	Left	Keratomalacia	10	78.5	8 days	Melting keratitis

mm=Millimeters, mm^2^=Millimeter square

### Ophthalmic examination

**Figure-1:**
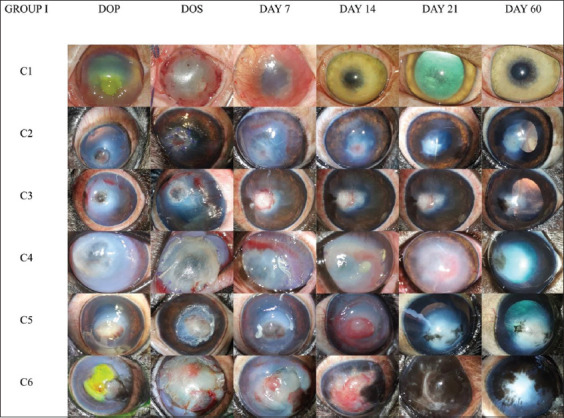
Gross photographs of corneas in Group I on the days of presentation, surgery, and post-operative days 7, 14, 21, and 60. C1: Extensive lesion of 13 mm diameter involving the whole cornea with visible prolapse of Descemet’s membrane that did not retain fluorescein dye but was disrupted at the center, exposing endothelium where the FDT was positive. The edges were healthy with progression of superficial blood vessels from the limbus. Graft uptake with intense neovascularization on post-operative day 7 resulted in absolute corneal clarity by day 14. C2: Axially located 6 mm diameter full-thickness defect with iris prolapse and localized edema. The cornea became hazy due to diffuse edema and vascularization by day 7, regained moderate clarity by day 14, and was completely clear by days 21 and 60, except for the central scar. C3: Full-thickness defect of 4 mm diameter with iris prolapse, localized corneal edema, and superficial vascularization. The cornea regained clarity by the end of the observation period, except for mild pigmentation. C4: Severely melting cornea with an 8 mm diameter full-thickness defect which stained fluorescein positive with dense edema and pannus all around the peri-limbal cornea. Corneal opacity due to edema complicated by stromal bullae retained up to day 14 post-grafting, which improved by day 21 and left a central scar remaining on day 60. C5: 6 mm diameter axially located full-thickness defect complicated by iris prolapse, melting edges, and miosis. The cornea became edematous by day 7 post-grafting and profusely vascularized by day 14, leaving considerable fibrosis and mild pigmentation by day 60. C6: Extensive chronic melting ulcer with irregular borders, diffuse corneal edema, and progression of pigmentation from the nasal limbal border, as the sclera was also heavily pigmented toward the medial canthus. Melting persisted on day 7, as well as complete opacity due to the central granulation tissue, profuse vascularity, and pigmentation on day 14. The cornea became extensively pigmented with an irregular scar at the center by day 21, which remained until the end of the observation period with pigmentation interspersed with a central scar. DOP=Day of presentation, DOS=Day of surgery.

**Figure-2:**
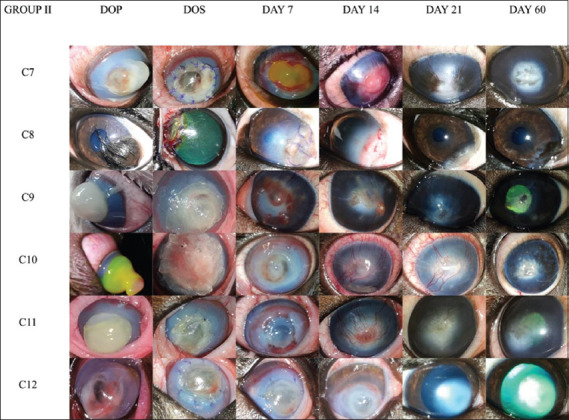
Gross photographs of corneas in Group II on the days of presentation, surgery, and on post-operative days 7, 14, 21, and 60. C7: Full-thickness corneal defect of 10 mm diameter with subsequent iris prolapse, melting edges, diffuse dense corneal edema, and neovascularization from the dorsal limbal border. The cornea remained moderately opaque post-grafting on days 7 and 14 due to intense vascularization and central granulation tissue. It became moderately clear by day 21 with a central scar and pigmentation but regained its clarity by day 60, except at the central region. C8: Congenital unilateral corneal dermoid extending at the temporal limbal border with scleral and corneal attachments, which were surgically removed by superficial keratectomy before grafting. The surgical site became opaque due to granulation and associated corneal edema by days 7 and 14, with scarring by day 21 that resolved by day 60, leaving mild pigmentation. C9: Staphyloma of 9 mm diameter with protruding endothelial layer stained positive with fluorescein dye. The entire cornea was edematous, and vascularization of the cornea was evident from almost all quadrants. Postoperatively, the cornea remained moderately opaque on days 7 and 14 due to intense vascularization, but regained clarity by day 21 which improved greatly by day 60, except for the central ghost vessel. C10: Twelve millimeter diameter full-thickness corneal defect with exposed endothelium and iris prolapse (1 cm above the corneal surface) staining positive with FDT. Corneal edema persisted up to day 7. Neovascularization was at its peak on day 14 and persisted through day 21 but resolved completely, leaving a central corneal scar, and mild melanosis compared to the initial defect size. C11: Staphyloma of 9 mm diameter with positive FDT, diffuse corneal edema, and pannus progression from the superior limbal border. Corneal edema persisted until day 7. The cornea was profusely neovascularized on day 14, eventually leaving a clear cornea except for the haze scar. C12: Chronic keratomalacia with a 10 mm diameter defect staining positive FDT, diffuse corneal edema, and circumferential superficial vascularization. The cornea gradually regained clarity through the observation period to leave a near-normal cornea by day 60. DOP=Day of presentation, DOS=Day of surgery.

The type and extent of the defect were determined based on the visual appearance, ophthalmic examination, and FDT. The photographs of corneas included in Group I and II on the day of presentation, grafting and subsequent post-operative reviews are depicted in Figures-1 and 2 respectively. Among 12 corneas studied, seven had full-thickness defects or staphylomas (58.33%), three had melting ulcers (25%), and one each (8.33%) had a descemetocele or keratectomy defect. The mean values of the lesion diameter were 7.83±1.33 mm in Group I and 9±1.09 mm in Group II. The area of lesion varied from 12.56 mm^2^ to 132.66 mm^2^ (mean 55.08±18.11 mm^2^) in Group I; and 32 mm^2^ to 113.04 mm^2^ (mean 71.53±10.82 mm^2^) in Group II. The mean values of aqueous tear production measured on the day of presentation and days 7, 14, 21, and 60 for both groups are presented in Table-3. The tear production was consistently high on the day of presentation and early post-operative review days due to possible corneal irritation but returned to the normal range by day 60 with complete resolution of the condition. All corneal defects except C8 were stained with Fluorescein dye on the day of presentation, and all corneas in Group I showed a negative FDT by day 7 post-grafting and on all subsequent observations. In Group II, epithelialization was not complete in C7, C10, and C11, resulting in a positive FDT on day 7 which became negative by day 14 post-grafting. The micro-organisms isolated from the cornea included *Staphylococcus aureus* (41.66%), *Staphylococcus intermedius* (16.66%), *Enterococci* spp. (16.66%), *Acinetobacter* spp. (8.33%), *Corynebacterium* spp. (8.33%), and *Pseudomonas aeruginosa* (8.33%). Intraocular pressure ranged from 11 mm Hg to 28 mm Hg, and all values were within the normal range. There was no significant difference in mean IOP measured within or between the groups during the observation period. The IOP measured on the central corneal scar was consistently higher than the IOP measured on the normal surrounding cornea, possibly due to fibrosis of the scars.

**Table-3:** The mean values of aqueous tear production (STT) and corneal grading factors including visual function score, corneal clarity, corneal edema, neovascularization of cornea and extent of pigmentation (mean±S.E) recorded on the day of presentation and on days 7, 14, 21, and 60 postoperatively in both Group I and Group II animals.

Group	Days	STT	Visual Function score	Corneal clarity	Corneal edema	Neovascularization of cornea	Extent of pigmentation
I	0	20.17±0.48	0.33±0.15	1.83±0.31	1.83±0.31	1.00±0.26	1.00±0.63
	7	20.50±0.67	0.38±0.20	2.00±0.37	1.67±0.33	1.67±0.33	0.83±0.65
	14	19.33±0.49	0.50±0.22	2.17±0.54	0.67±0.42	1.50±0.43	1.33±1.14
	21	18.17±0.60	0.72±0.18	2.83±0.40	0.00±0.00	0.17±0.17	3.83±2.87
	60	15.83±0.40	0.88±0.11	3.00±0.26	0.00±0.00	0.17±0.17	2.50±1.93
II	0	21.83±0.87	0.17±0.17	1.50±0.50	2.50±0.50	0.67±0.21	0.00±0.00
	7	21.50±0.88	0.17±0.17	1.50±0.34	2.17±0.31	2.17±0.40	0.17±0.17
	14	20.00±0.68	0.61±0.18	1.83±0.31	0.50±0.34	2.33±0.33	0.17±0.17
	21	18.50±0.72	0.89±0.07	2.67±0.21	0.00±0.00	0.83±0.17	0.67±0.67
	60	17.17±0.70	1.00±0.00	3.33±0.21	0.00±0.00	0.17±0.17	0.33±0.33

STT=Schirmer tear test

### Grading of cornea

**Figure-3:**
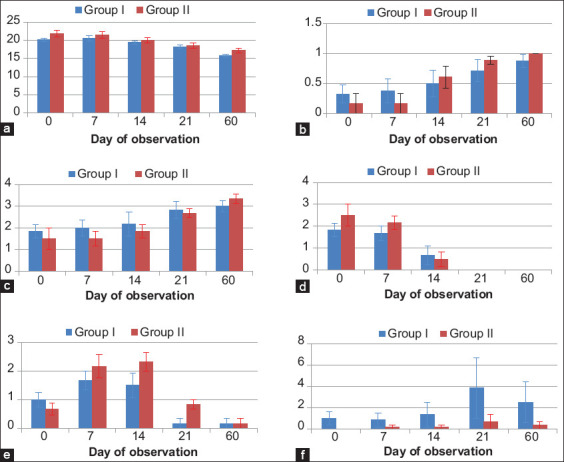
a) Schirmer Tear Test: Though there was no variation mean tear production till day 14, significant difference was observed from day 21 in both Group I and II. The mean value of tear production did not show any significant difference between the groups. b) Visual Function: Significant variation in mean visual function score was observed early by day 21 in Group II animals compared to 60 days in Group I (p<0.05). No significant variation could be observed between groups. c) Corneal Clarity: Significant variation in mean corneal clarity was observed early by day 21 in Group II animals compared to 60 days in Group I (p<0.05). No significant variation could be observed between groups. d) Corneal Edema: Though there was no variation in corneal edema till day 7, significant difference was observed from day 14 in both Group I and II. The mean value of corneal edema did not show any significant difference between the groups. e) Neovscularization of Cornea: Significant variation in mean neovascularization was observed between day 0, 7 and 14 and day 21 in both the groups. The mean values of neovascularization did not show any significant variation between the groups except on day 21 (p<0.05). f). Corneal Pigmentation: There was no significant difference in mean values for the extent of corneal pigmentation within the groups or between the groups during the observation period. Minimum pigmentation in MMC treated Group II was a consistent finding.

The mean values of the visual function score, corneal clarity, corneal edema, neovascularization of cornea, the extent of pigmentation, and corneal scarring in Group I and Group II are represented in Table-3 and Figure-3. Visual function scores were 0.17±0.17 in Group II on the day of presentation, improving to 1.0±00 by the end of the observation period. In Group I, they ranged from 0.33±0.15 on the day of presentation to 0.88±0.11 by the end of the observation period. The mean value of the corneal clarity score improved from 1.83±0.31 on the day of presentation to 3±0.26 on day 60 in Group I; and from 1.5±0.34 to 3.33±0.21 in Group II. Significant variation in mean visual function score and corneal clarity was observed early by day 21 in Group II corneas, compared to after 60 days in Group I (p<0.05). In most cases, dense and diffuse corneal edema at presentation persisted on day 7, decreased significantly by day 14, and resolved completely by day 21 in both the groups. The mean value of corneal edema did not show any significant difference between groups during the observation period. Neovascularization was at its initial stage on the day of presentation, with a mean value of 1.00±0.21 in Group I and 0.67±0.21 in Group II, peaked by day 7 in Group I (1.67±0.33) and day 14 in Group II (2.33±0.33), and disappeared by day 60 (0.17±0.17) in both the groups. The extent of pigmentation was graded from 0 to 24. C1, C2, C4, C9, C10, C11, and C12 had no corneal pigmentation on the day of presentation and had none develop during treatment and the observation period. C3 showed limbal melanosis toward the nasal quadrant on the day of presentation, which progressed toward the center of the cornea during wound healing. In C6 and C7, corneal pigmentation developed by day 21 post-operative but resolved completely in C7 by the end of the observation period. There was no significant difference in mean values for the extent of corneal pigmentation within groups or between groups during the observation period. Corneal scarring was evaluated on the last day of the examination, with scores reported in Table-1. No major complications were encountered except an alteration in the shape of the iris in C2 and C3, and extensive melanosis and scarring in C6 that did not impaired vision.

## Discussion

Corneal wound healing is a complex phenomenon that includes activation of keratocytes, differentiation into fibroblasts, proliferation, and migration to the site of injury to re-populate the area that had already been depleted of keratocytes, followed by the production of collagen, glycosaminoglycans, and ground substance to repair the defect. The rationale for choosing decellularized bovine omentum as an extracellular matrix scaffold was based on its abundance of cell signaling factors essential for cell adhesion, migration, proliferation and differentiation, and growth factors. The decellularization and defatting of bovine omentum preserve the three-dimensional architecture of connective fibers, collagen, reticular, elastic fibers, and glycosaminoglycans essential for wound healing [8]. The trans-differentiation of keratocytes to myofibroblasts involved in cellular matrix remodeling will result in disoriented ECM rich in Types III and IV collagen, causing loss of corneal transparency. Myofibroblasts cause corneal fibrosis, though they are essential components of corneal healing. Suppression of myofibroblast formation and anti-fibroblast and anti-mitotic effects of MMC through enhanced apoptosis and depressed cellular proliferation has been demonstrated [9]. The present study was undertaken to evaluate the ability of decellularized bovine omentum as an ECM scaffold to enhance healing, and MMC as an antifibrotic agent to minimize the complication of scarring during corneal healing.

Brachycephalic breeds were over-represented (11 animals), accounting for 91.76% of all cases, which is likely due to the brachycephalic conformation of the head with lagophthalmos, macropalpebral fissure, insufficient painting of tear over the cornea, decreased corneal sensitivity, medial canthal trichiasis, and entropion [13]. All of the animals affected were below 3 years of age, with 45.45% below 1 year, 36.36% from 1 to 2 years, and 9% from 2 to 3 years of age. The incidence, was remarkably high in younger age groups, potentially because these animals are in the most active period of their lives [14]. There was an equal representation of both sexes, suggesting insignificance in the sex of affected animals in this small treatment group [15].

The size of the defect was considerably larger in Group II compared to Group I. As a result, the interval from initiation of treatment and stabilization of the corneal stroma to complete epithelialization of the defect was longer in Group II than in Group I. The difference in duration of ulcer healing in different groups may be attributed to different average defect sizes [1]. Tear production was consistently high on the day of presentation and at early post-operative review days, and returned to the normal range by day 60 with complete resolution of the condition. Higher values on initial post-operative reviews may have been due to irritation caused by the graft, as well as the suture material. All corneas except one showed positive FDT on the day of presentation, as most were full-thickness defects and the endothelium is lipophilic [16]. Graft healing time was assessed by the time taken for complete ulcer re-epithelialization, indicated by negative FDT of the grafted cornea [4]. All corneas in Group I showed a negative FDT by day 7 post-grafting, indicating complete epithelialization, whereas three out of six corneas were FDT positive in Group II at the same time point. However, all corneas in Group II showed negative FDT on day 14 and remained so on subsequent days. This indicated delayed epithelialization of the cornea when MMC was used and may be attributed to the cytotoxic effect of the drug. MMC has been shown to significantly inhibit the multiplication of corneal epithelium, keratocytes, and endothelium due to its anti-mitotic effect in a trial with *in vitro* culture of canine stromal fibroblast cells [9]. *Staphylococcus* spp., was identified as the most frequent (58.32 %) bacterial pathogen causing canine infectious keratitis. *P. aerugenos*a infected corneas had persistent chronic melting until post-operative day 7 which are reported to be capable of destroying the entire corneal epithelium and stroma, making this pathology difficult to control [17]. Comparatively higher values of IOP in Group I may be attributed to an increase in the corneal stromal thickness due to fibrosis and changing in curvature as a result of wound healing.

### Corneal grading

Eight out of 12 eyes were visually impaired on the day of presentation due to extensive corneal defects and dense corneal edema with negative menace reflex, cotton ball test, and undetectable PLR. Visual function gradually improved through days 7 and 14, and all animals had vision by day 21 that was maintained through day 60. All animals had positive visual function by day 60 as assessed by menace reflex [3]. The visual outcome achieved in the study was superior to the 87% vision achieved when ACell Vet™ (USA) was used as a biomaterial graft, 93% achieved with an acellular submucosal graft covered by conjunctival graft, 93% with a conjunctival pedicle graft and corneoscleral transposition grafts, and 83.33% with a porcine cholecyst derived collagen scaffold graft [7,13,18]. The factors impeding corneal clarity were corneal edema on the day of presentation and in the early post-operative period, as well as pigmentation and corneal scarring toward the end of the observation period. Corneal clarity gradually improved through the observation period, achieving a completely clear cornea in one out of six cases in Group I and three out of six cases in Group II, indicating that MMC is effective in restoring corneal clarity.

The reduction in pigmentation and scarring toward the end of the observation period may be attributable to the corticosteroid therapy that markedly decreased vascularization and, thereby, pigmentation [19]. A substantial improvement in corneal clarity could be appreciated at the end of the observation period in six out of 12 corneas from brachycephalic breeds, other than the Chinese Pugs. Most of the corneas that failed to regain clarity were of Pugs [3]. Intense corneal edema persisted in nine cases of full-thickness defect on day 7, which may be attributed to damage to Descemet’s membrane in full-thickness injury and graft opacification due to continuous entry of aqueous humor to the graft [11]. Corneal neovascularization is the primary step for integration of the graft into the corneal stroma. The number of blood vessels present initially was enhanced by surgery and amplified during graft integration due to activation by growth factors present both within the graft and the cornea [7]. Intense neovascularization developed from almost all quadrants with fine, repeatedly branching vessels in large corneal defects compared to less neovascularization observed with smaller diameter defects. There was a significant variation in the neovascularization of cornea between the two groups and within the groups between weeks. This may have been due to the large size of the defect and the resulting delayed wound healing in the MMC group.

Three corneas in Group I and one cornea in Group II (33.33%) developed corneal pigmentation as a complication of the healing ulcer, which may be attributed to brachycephalic pigmentary keratitis syndrome; an initially pigmented cornea may predispose to worsening of the pigmentation after corneal grafting, especially in cases of extensive corneal lesions such as descemetoceles and staphylomas [20]. When the corneas were graded for fibrosis and scarring, 33.33% obtained “grade 0” with an almost undetectable corneal opacity (one animal in Group I and three animals in Group II), and 16.66% obtained “grade 1” with minimal stromal opacity and clear visualization of the anterior segment through the grafted site. In Group II alone, 50% of the corneas obtained grade “0” with an undetectable corneal scar, which may be attributable to the anti-fibrotic effect of MMC [9].

## Conclusion

The data presented here indicate that the decellularized bovine omental ECM scaffold enhanced corneal healing by providing a medium for recellularization; and single time intra-operative application of MMC considerably reduced the incidence of corneal fibrosis and scarring to restore clarity.

## Authors’ Contributions

AST performed the experiment, collected the data, and drafted the manuscript. AS designed and supervised the experiment, reviewed the data analysis, and revised the manuscript. KMD, VNV, LMP, and CBD supervised the experiment and revised the manuscript. All authors read and approved the final manuscript.
